# Blended teaching mode based on small private online course and case-based learning in analgesia and sedation education in China: a comparison with an offline mode

**DOI:** 10.1186/s12909-023-04839-4

**Published:** 2024-01-04

**Authors:** Shu Li, Longxiang Su, Ran Lou, Ying Liu, Hua Zhang, Li Jiang, on behalf of the Chinese Analgesia and Sedation Education and Research (CASER) group

**Affiliations:** 1 Department of Critical Care Medicine, Peking University People’s Hospital, Beijing, China; 2 Department of Critical Care Medicine, State Key Laboratory of Complex Severe and Rare Diseases, Peking Union Medical College Hospital, Chinese Academy of Medical Science and Peking Union Medical College, Beijing, China; 3 Department of Critical Care Medicine, Xuanwu Hospital, Capital Medical University, Beijing, China; 4 Department of Critical Care Medicine, Affiliated Hospital of Guizhou Medical University, Guizhou, China; 5 Research Center of Clinical Epidemiology, Peking University Third Hospital, Beijing, China

**Keywords:** SPOC, CBL, PAD management, Postgraduate medical education

## Abstract

**Background:**

Standardized training for pain, agitation-sedation, and delirium (PAD) management is urgently needed for Chinese intensivists’ continuing education. Since 2020, because of the COVID-19 pandemic, the Chinese Analgesia and Sedation Education and Research (CASER) group has used an online blended teaching mode based on a small private online course (SPOC) and case-based learning (CBL). This study evaluated whether an online blended teaching mode has similar effects on PAD management training when an offline mode cannot be used.

**Materials and methods:**

Since 2020, the CASER group has provided offline training and online SPOC&CBL training three times each, targeting intensivists and ICU nurses in China. All participants were divided into an offline group and SPOC&CBL group. A final examination was offered in each training session to assess the students' mastery of professional knowledge. Teachers’ and students’ perceptions regarding the online SPOC&CBL mode were evaluated through questionnaires.

**Results:**

Of all participants (*n* = 117), 106 completed all examinations and questionnaires. Most participants were aged 31–40 years (53, 50.0%), had an academic degree (60, 56.6%), and worked in a tertiary hospital (100, 94.34%). We assessed the learning effect on participants from two aspects: theory and clinical practice. There was no significant difference between the SPOC&CBL and offline groups in terms of theoretical, case analysis, and total scores (*p* > 0.05). In terms of the participants’ perceptions regarding the SPOC&CBL mode, 91.5% considered the online mode to be a useful and accessible alternative to improve knowledge and skills. A total of 95.7% of the participants believed that they could interact well with group members, and 87.2% believed that they had a good degree of participation. Of these participants, 76.6% believed that they had received valuable learning resources. All instructors believed that the SPOC&CBL mode was more flexible than the offline mode in terms of teaching time and location, and they were all willing to carry out training with the SPOC&CBL mode.

**Conclusion:**

Compared to the offline mode, the SPOC&CBL mode can also enhance participants’ knowledge and skills and meets their expectations. Therefore, an online mode can be considered a potential method in PAD management education in China.

## Introduction

Pain, agitation-sedation and delirium (PAD) management is an integral part of intensive care [1, 2]. However, inappropriate PAD management, such as analgesia or sedation that is too deep or too light or undetected delirium, may have serious adverse effects on patient prognosis [3, 4]. Therefore, comprehensively mastering theoretical knowledge of PAD management and using it proficiently in clinical practice is an important part of optimizing treatment. To promote PAD management training, the Chinese Analgesia and Sedation Education and Research (CASER) group was formed with the support of the Chinese Association of Critical Care Physicians (CACCP). In 2020, the CASER team carried out several offline training sessions that achieved good results and improved the medical staff's theoretical knowledge and clinical capability of PAD management.

In the second half of 2020, because of the COVID-19 pandemic, the CASER group had to change the mode from offline to online training. Small private online courses (SPOCs) are a popular teaching method in education. In SPOCs, the number of participants is limited, and the participants must meet certain criteria so that the content of the course can be more accurate, which can effectively help students supplement the knowledge that needs to be improved [5]. However, SPOCs are not popular in medical education because clinical scenario simulation training is difficult to carry out in online mode. Fortunately, the experience of the CASER group in offline training modes showed that the ability to apply PAD management theory in actual clinical work can be improved by discussing and analysing actual cases. Moreover, to understand students' concerns about the online training mode, we conducted a prequestionnaire with our previous students. The results showed that more than 85% of the participants believed that engagement and interactivity are areas that need improvement in online trainings. Therefore, the CASER group has used a blended teaching mode based on a SPOC and case-based learning (CBL) in PAD management training. CBL consists of the analysis of medical records with the aim of duplicating the actual clinical scenario and prompting students to identify and develop new areas of learning, which can be very effective in promoting students to combine theory with clinical practice and improve their clinical problem-solving abilities [6, 7]. Moreover, some activities have been optimized to promote interaction. In this study, we conducted tests and questionnaires for instructors and students to compare the effects of the SPOC&CBL and offline teaching modes on PAD management training.

## Materials and methods

### Design of courses

The project management team consisted of clinicians, medical teachers, statisticians, pedagogists, and a graphic designer. The teaching team consisted of four critical care medicine instructors who are also intensivists with more than 15 years of teaching experience and clinical work experience. The project management team was responsible for 1) building the overall training structure, including training objectives (improving the theoretical knowledge and clinical practice ability in PAD management) and specific content (principles of analgesia and sedation treatment, drug selection, assessment scales, etc.), understanding the participants’ needs, and evaluating the effectiveness of the training; and 2) organizing the members of the teaching team to conduct training and prepare centralized lessons in the SPOC&CBL teaching mode. The teaching team was responsible for making course-related multimedia materials, collecting and arranging course-related literature and materials, organizing clinical cases, and teaching during each training session.

For offline courses, we recruited students from different hospitals to complete face-to-face courses at a specific time and place. First, the teacher explained the course content. Then, the students were divided into several groups, and the groups discussed 10 actual clinical cases. After the discussion, an examination was conducted. The number of people in each course was limited to 30.

The SPOC&CBL courses include the following aspects. Before the training, the project management team collected information from the registered students, including their major, education level, seniority, medical institution and position, and conducted a basic assessment of the students to ensure that they met the criteria for participating in the course. In the theoretical knowledge review and learning section, the online live broadcast platform was used, and the teaching team conducted training in various ways, such as by using PowerPoint slides, multimedia materials, and reference materials. Afterwards, in the discussion and clinical practice part, the students were divided into several groups, with five to six participants per group. A simulation scene was set up based on 10 classic cases, and the groups discussed the cases. The instructors guided the students in applying theoretical knowledge to practical clinical scenarios during the discussion. Finally, the instructors summarized the instruction through the live broadcast platform, provided feedback on the performance of each group, instructed the participants to review the material after class, and assessed the professional theoretical knowledge and clinical application capability of the participants by tests.

The training objectives and content were similar in the offline and SPOC&CBL training modes. To enhance interaction in the online SPOC&CBL mode, we asked the teachers to interact with each student. In the discussion, every student was required to make a speech. For the final certificate, the students who participated in the discussion and actively asked questions were graded separately, and the students with high scores received an A-level certificate.

### Participants and study setting

Since 2020, the CASER group has held PAD management training regularly. The training class included physicians and nurses working in intensive care units (ICUs) nationwide as participants, and no more than 20 trainees were recruited for each session. In the first half of 2020, three offline teaching sessions were held, and three online SPOC&CBL training sessions were held from the second half of 2020 to April 2022.

### Evaluating the effect of each teaching mode

The students took the exams after completing the courses. The test included six theoretical questions about analgesia, sedation and consciousness assessment and six questions on clinical case analysis. The two groups of students complete the same test paper for the postclass test. In addition, the students were taught by the same teachers, who also completed the grading and interpretation of the test paper. Therefore, the course content and test paper difficulty were identical between the two groups of students.

From July to October 2022, electronic questionnaires were delivered to six instructors and all the participants to evaluate their perceptions of the SPOC&CBL blended teaching mode and the teaching effect. The questionnaire for instructors consisted of items regarding the purpose, significance, sponsoring organization (CACCP), ethics statement and perception of the training effect of the SPOC&CBL mode. The questionnaire for participants consisted of three parts: 1. a preface, which explained the purpose, significance, sponsoring organization, and ethics statement; 2. a general information section, including items on the sex, age, educational level, professional and technical title, working department, and number of years of working experience of the participants; and 3. a section on the participants’ perceptions of the training effect of the SPOC&CBL mode.

The questionnaire was organized and completed by the expert group according to the purpose of this research. The members of the expert group included six doctors (with more than 15 years of work experience) from comprehensive ICUs in different provinces, two nurses and one medical statistician. The pilot testing of the questionnaire was completed by 12 trainees who participated in the training, and various data were collected, including the layout, structure, attractiveness, question setting, respondent tolerance, and respondent understanding of the questions. SPSS software 22.0 was used for content validity analysis, and the Cronbach’s alpha value was 0.793, indicating that the questionnaire was effective.

### Statistical analysis

SPSS software (version 22.0) was used to analyse the data. Categorical variables are expressed as percentages and were compared by the chi-square test or Fisher’s exact test. Continuous data are reported as medians with interquartile ranges and were compared using the unpaired Mann‒Whitney U test. Statistical significance was set at *p* < 0.05.

## Results

### Characteristics of the participants

Questionnaires were delivered to all 117 trainees who participated in the training. A total of 106 valid questionnaires from trainees were returned. Among the 106 participants, there were 54 males (50.94%) and 52 females (49.06%). Most participants were aged 31–40 years (53, 50.0%). There were 94 physicians (88.68%) and 12 nurses (11.32%). Most participants had master’s degrees (60, 56.6%). There were at most 55 (51.89%) participants with intermediate titles (attending physician), 30 with senior titles (leading a medical team, 28.3%), and at least 21 with junior titles (resident physician, 19.81%). Most of the participants worked in tertiary hospitals (100, 94.34%). There were 72 (67.92%) participants who worked in general ICUs and 34 (32.08%) participants who worked in specialized ICUs. In terms of the number of working years, 18 (16.98%) participants had worked for less than 5 years, 29 (27.36%) had worked for 5–10 years, and 59 (55.66%) had worked for more than 10 years. The 106 participants were divided into groups according to the different teaching methods, and there was no significant difference in the basic characteristics between the two groups (Table 1).

**Table 1:** Comparison of the baseline characteristics of the participants in the SPOC&CBL and offline groups

Characteristic	SPOC&CBL group (*n* = 47)	Offline group (*n* = 59)	*p* value
Age range (years), *n* (%)			0.950
< 25	1 (2.1)	1 (1.7)	
25–30	7 (14.9)	12 (20.3)	
31–40	25 (53.2)	28 (47.5)	
41–50	12 (25.5)	16 (27.1)	
> 50	2 (4.3)	2 (3.4)	
Sex, n (%)			0.248
Male	27 (57.4)	27 (45.8)	
Female	20 (42.6)	32 (54.2)	
Academic degree, *n* (%)			0.122
Associate’s degree^a^	1 (2.1)	5 (8.5)	
Bachelor’s degree	7 (14.9)	17 (28.8)	
Master’s degree	30 (63.8)	30 (50.8)	
Doctoral degree	9 (19.1)	7 (11.9)	
Profession, *n* (%)			0.415
Doctor	43 (91.5)	51 (86.4)	
Nurse	4 (8.5)	8 (13.6)	
Professional title level, *n* (%)			0.971
Junior	9 (19.1)	12 (20.3)	
Intermediate	25 (53.2)	30 (50.8)	
Senior	13 (27.7)	17 (28.8)	
Department, *n* (%)			0.699
General ICU	31 (66.0)	41 (69.5)	
Specialized ICU	16 (34.0)	18 (30.5)	
Number of working years, *n* (%)			0.834
< 5	7 (14.9)	11 (18.6)	
5–10	15 (31.9)	14 (23.7)	
11–15	14 (29.8)	20 (33.9)	
16–20	4 (8.5)	7 (11.9)	
> 20	7 (14.9)	7 (11.9)	
Hospital type, *n* (%)			0.435
Tertiary hospital	45 (95.7)	55 (93.2)	
Secondary hospital	2 (4.3)	2 (3.4)	
Private hospital	0	2 (3.4)	

^**a**^ Students who graduate from a two-year junior college in medicine can receive an associate’s degree

*SPOC* Small private online course. *CBL* Case-based learning. *ICU* Intensive care unit

### Perception of teachers regarding the SPOC&CBL mode

**Fig. 1:**
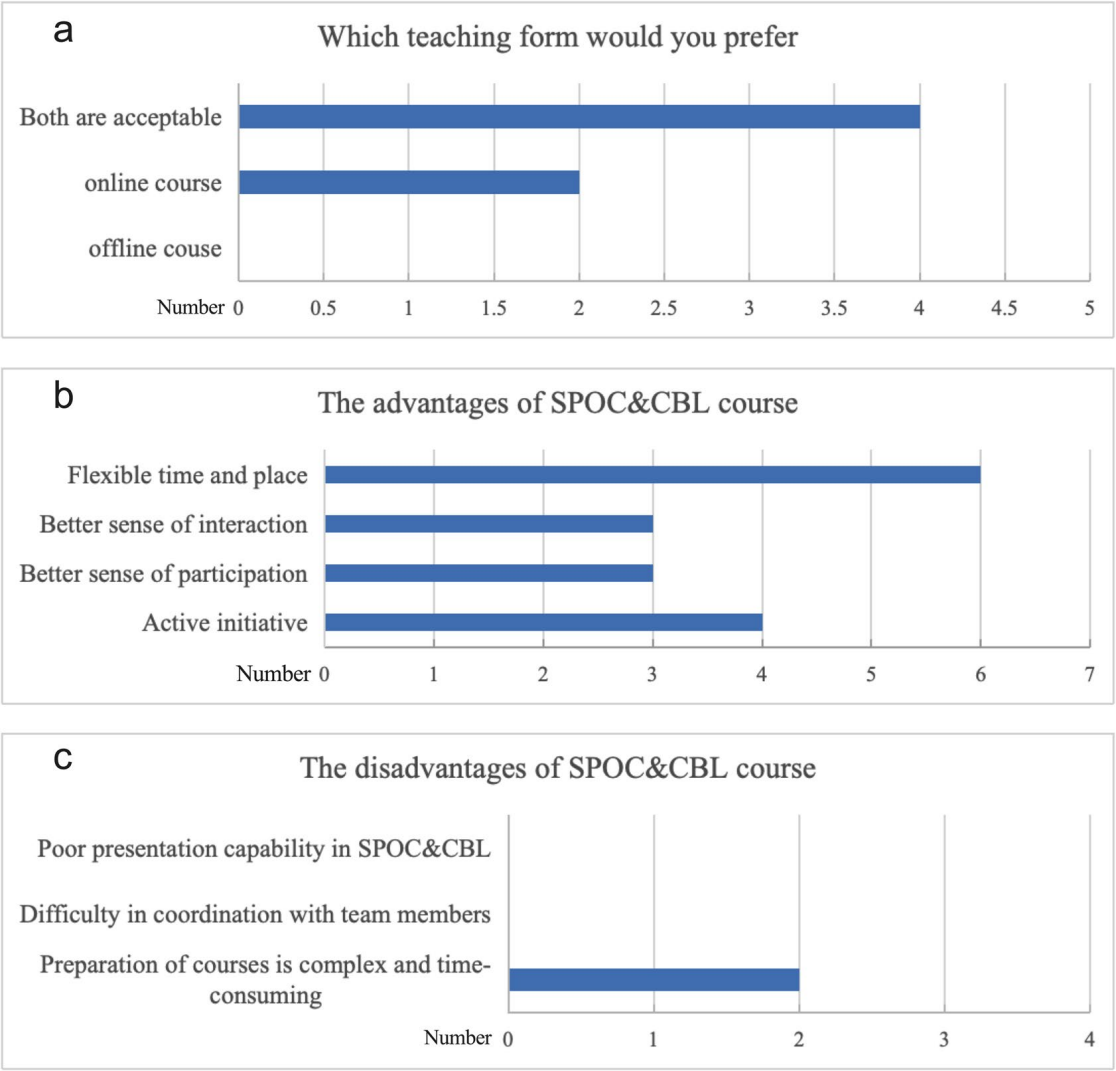
Perception of teachers regarding the SPOC&CBL mode. SPOC, small private online course. CBL, case-based learning

The results of questionnaires from six instructors showed that all six were willing to carry out online courses, and two of them preferred the SPOC&CBL mode to the offline mode (Fig. 1a). In terms of the advantages of the SPOC&CBL mode, all instructors believed that it was more flexible than the offline course mode in terms of teaching time and location. Three instructors believed that the SPOC&CBL mode provided students with a more engaging and interactive scenario. Four instructors believed that the online mode was more conducive to stimulating students to learn actively (Fig. 1b). Two instructors believed that the preclass preparation required more work and took longer in the SPOC&CBL mode than in the offline mode (Fig. 1c).

### The influence of the SPOC&CBL mode on the test scores of participants

According to the content and focus of the training, we assessed the learning effect on the participants from two aspects: theory and clinical practice. The theoretical part and the clinical part each consisted of 50 points, with a total score of 100 points. The results showed that there was no significant difference between the SPOC&CBL group and the offline group in terms of theoretical, case analysis, and total scores (*p* > 0.05) (Table 2).

**Table 2:** Comparison of the test scores between the SPOC&CBL group and offline group

	SPOC&CBL group (*n* = 47)	Offline group (*n* = 59)	*p* value
Total score, median (P25, P75)	50.00 (41.67, 58.33)	50.00 (33.33, 66.67)	0.992
Theory test score, median (P25, P75)	25.00 (16.67, 33.33)	25.00 (16.67, 41.67)	0.863
Case-analysis test score, median (P25, P75)	25.00 (16.67, 33.33)	25.00 (16.67, 33.33)	0.475

*SPOC* Small private online course. *CBL* Case-based learning

#### Perceptions of students regarding the SPOC&CBL mode

**Fig. 2:**
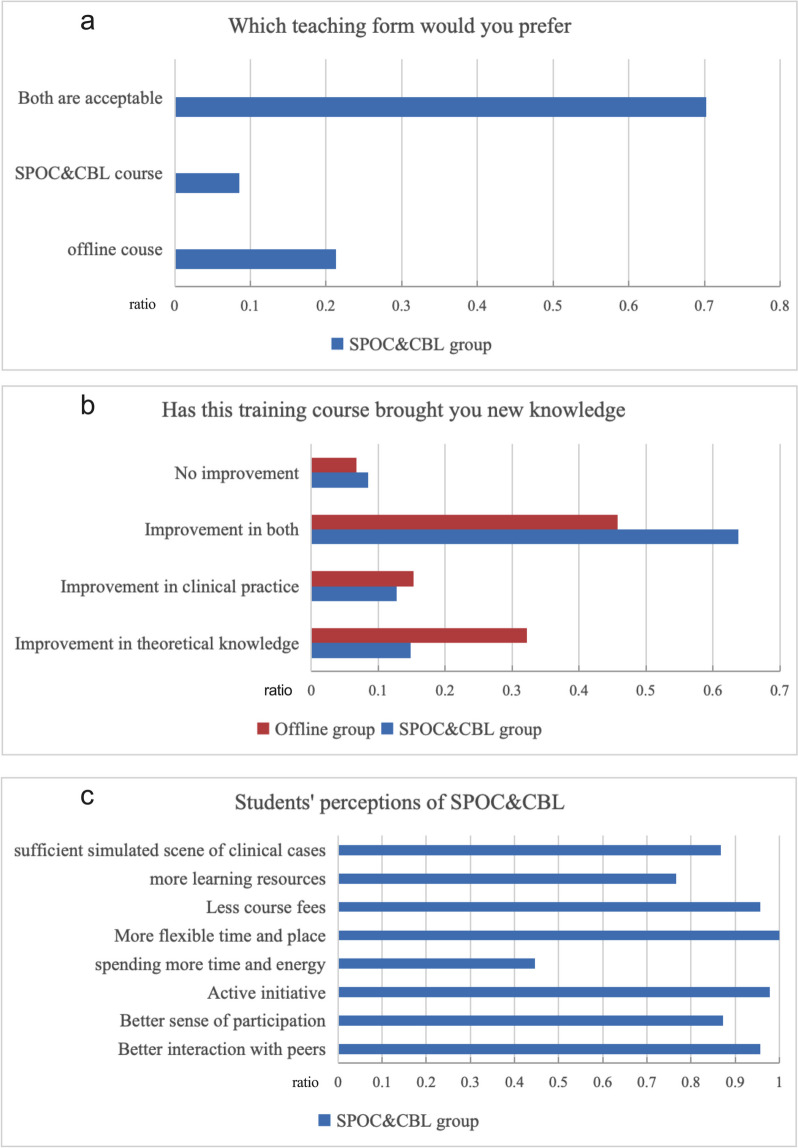
Perceptions of students regarding the SPOC&CBL mode. SPOC, small private online course. CBL, case-based learning

In addition to understanding the learning effect on the students through objective tests, we also studied the perceptions of the participants through the questionnaires. In terms of the participants’ acceptance of the online course, the results showed that 72 (67.92%) students preferred the online mode or both the online and offline modes (Fig. 2a).

In terms of learning benefits, 93.3% of the offline group believed that they had benefited in terms of theoretical knowledge, case application or both. A total of 91.5% of the participants in the SPOC&CBL group believed that they had benefited in terms of theoretical knowledge, case application, or both (Fig. 2b).

We investigated the opinions of the participants in the SPOC&CBL group regarding the advantages and disadvantages of the online mode. In terms of advantages, 95.7% of the participants believed that they could interact well with group members during the course, and 87.2% believed that they had a good degree of participation in the class. A total of 97.9% of the participants thought that the learning initiative was well stimulated, and 76.6% believed that they had received valuable learning resources throughout the course. A total of 86.8% of the participants believed that the clinical case simulation scenario was sufficient. Flexibility of time and location and the low cost of courses were also advantages recognized by almost all participants. In terms of disadvantages, 44.7% of the participants thought that it took more time and energy to participate in training with the SPOC&CBL mode (Fig. 2c).

## Discussion

The results of this study showed that the blended SPOC&CBL mode is effective in PAD management training in China compared with the offline mode. According to the analysis of the participants' objective test scores and subjective perceptions, the instructional effectiveness of the SPOC&CBL mode was similar to that of the offline mode. Moreover, for the new blended teaching mode, instructors and participants had a good degree of acceptance. The characteristics of a more flexible time and place and lower costs for the online mode made it easier for participants from all over the country to participate in the training. It also allowed the CASER group to promote PAD management training in China in an orderly and in-depth manner in the context of the COVID-19 pandemic.

PAD management for critically ill patients has been recognized for more than 10 years in China. Judging from the increasing number of reports and discussions in conferences regarding critical illness, the importance of PAD management is recognized by intensivists in China [8–10]. However, there are still many deficiencies in the mastery of specific theoretical knowledge and clinical practice [11–13]. Exploring different teaching forms, improving teaching effectiveness, and conducting consistent training on PAD management for intensivists across the country are the core content of the CASER group's work. We have achieved good results with previous offline trainings, and the majority of participants benefit from the trainings. However, with the expansion of the training program, we also realize that the training mode still needs further development. PAD management training is not included in the medical undergraduate and graduate education curriculum but forms part of the continuing education content for critical medicine. The organization of offline teaching modes is obviously not as convenient and feasible as that of online teaching modes. In addition, the COVID-19 pandemic has an unprecedented impact on global public health and affected the medical education sector [14]. Therefore, in later training, we switched from the offline training mode to the online training mode.

Online modes are not as popular in Chinese medical education as in education in other fields. There are many practical clinical operations in medical training that are difficult to conduct with online modes. However, in PAD management training, discussing and analysing actual clinical cases can promote participants' ability to translate theoretical PAD knowledge into clinical treatment. Moreover, our preliminary research on offline training found that most of the intensivists in China had a clear understanding of the concept of PAD management, but the actual application in clinical cases was not ideal. Therefore, in our PAD management training, clinical practice was the focus of training. We used an online CBL mode in this study. Based on typical clinical cases, participants participated in group discussions to explore the optimal PAD management plan, which was conducive to their ability to solve clinical practical problems [15, 16].

PAD management training includes much content, and there are many levels of difficulty in the course [17]. The PAD management training in this study was mainly aimed at doctors with little experience in PAD management. This course was chosen voluntarily by the intensivists. Before the course started, the outline and content was announced, and the difficulty of the course was explained. Therefore, when students chose courses, they were informed of whether they were suitable according to the contents of the course. Otherwise, we choose an SPOC to carry out online training. The SPOC was smaller and developed for a targeted audience and was, therefore, more suited to the educational needs of our participants. Notably, PAD management has not been promoted in China for a long time, and the training level in different regions varies greatly. Therefore, the working years and age of the trainees were not related to PAD management experience.

All the instructors in this study were willing to teach using the SPOC&CBL mode, and two even preferred to teach with the SPOC&CBL mode only. The flexibility of time and place were undoubtedly outstanding advantages of the SPOC&CBL mode compared to the offline mode. To minimize the shortcomings of less interaction between instructors and students and strengthen the role of instructors in the online SPOC&CBL mode, instructors provided the theoretical knowledge training live, and they could interact with participants at any time during this part. In CBL group discussions, the instructors also participated in and guided the participants' discussions online. As a result, our instructors believed that in the SPOC&CBL mode, students' learning initiative and participation were also good, and students' participation in group discussions was better. Of course, in this mode, instructors had more work content, and two instructors thought that the SPOC&CBL mode consumed more energy and time than the offline mode. It has been reported that teachers believe that they spend considerable time and energy preparing SPOCs [18]. Teachers using the SPOC&CBL curriculum need professional knowledge, substantial clinical experience, and excellent leadership capability. They need to update the teaching concepts and learn new teaching theories and put them into use. Additionally, they must develop and design an education portal and courses using more information technology. In the future, the relevant staff of the CASER group will further optimize the preparation of training, teaching, and after-class summary processes to help instructors provide online trainings more easily.

The effectiveness of online and offline teaching in medical education remains uncertain. Evaluations have failed to lead to consistent conclusions [19–21]. In a systematic review and meta-analysis, whether online learning, when compared to offline learning, could improve the learning outcomes of undergraduate medical students was evaluated. Overall, online learning was at least as effective as offline learning [21]. The results of our study also showed that the effect of the online mode was no worse than that of the offline mode. Since the purpose of the training and the main content were the same in both the offline and SPOC&CBL groups, we tested all the participants using the same test to evaluate the learning effect. The results showed that there was no difference in the scores of the two groups. Therefore, participants’ benefits from the course were not affected by the learning mode. In the Netherlands, a study was conducted to evaluate an SPOC according to interns’ first impressions and satisfaction measures regarding the SPOC [22]. The evaluation showed that the SPOC was a useful and accessible addition to the clinical learning environment, providing an alternative opportunity for the interns to improve their knowledge and skills. However, it appeared that the interns were not satisfied with the collaboration and relatedness aspects of the SPOC, particularly the web-based interaction and peer feedback. The results of our study also showed that participants' perceptions and acceptance of the SPOC&CBL teaching mode were also relatively good. More than 90% of the participants believed that they benefited from the course. They interacted well with other peers in the course and obtained a rich learning experience. The reason for these differences may be that teachers and students communicated and provided feedback in the online course, strengthening the interactions between teachers and students. Other studies also confirmed that students prefer teacher feedback over peer feedback [23]. Moreover, our results showed that all participants felt that the flexibility of class time and location was conducive to participating in the training. Our participants came from hospitals all over the country, and the online mode obviously saved them travel and accommodation costs.

## Conclusion

An online blended teaching mode based on an SPOC and CBL is a good educational method for PAD management training when students cannot access an offline course. By optimizing the role of CBL in the online mode and strengthening the discussion and analysis of actual cases, the students’ ability to solve practical clinical problems was enhanced. Our innovative exploration provides a new perspective for curriculum reform in clinical medicine. It also offers optimal propagation of information in a cost-effective way and meets students’ expectations for training.

## Data Availability

The datasets used and/or analysed during the current study are available from the corresponding author upon reasonable request.

## Abbreviations

CASER: Chinese Analgesia and Sedation Education and Research
PAD: Pain, agitation-sedation, and delirium
SPOC: Small private online course
CBL: Case-based learning
CACCP: Chinese Association of Critical Care Physicians
ICU: Intensive care unit
